# Ranizurel safety evaluation in real-world -(RaSER) study

**DOI:** 10.1016/j.ajoc.2022.101358

**Published:** 2022-02-02

**Authors:** Ashish Sharma, Jayshree Arunaprakash, Atheeshwar Das, Ashraya Nayaka, Nilesh Kumar, Nikulaa Parachuri, Baruch D. Kuppermann

**Affiliations:** a Lotus Eye Hospital and Institute, Avinashi Road, Coimbatore, TN, India; b Dr Agarwals Eye Hospital, Coimbatore, TN, India; c Medicare Eye Hospital & Laser Vision Center, Chennai, TN, India; d The Eye Foundation, Coimbatore, TN, India; e Madhavi Netralaya Ara, Bihar, India; f Sankara Eye Hospital, Coimbatore, TN, India; g Gavin Herbert Eye Institute, University of California Irvine, Irvine, CA, USA

**Keywords:** RanizuRel, Biosimilar, Real world, Early experience, Anti-VEGF, **DME**, Diabetic macular edema, **CRVO**, Central retinal vein occlusion, **n-AMD**, neovascular age related macular degeneration, **IRF**, Intra-retinal fluid, **NSD**, Neurosensory detachment, **CME**, Cystoid macular edema, **SRF**, Sub retinal fluid

## Abstract

**Purpose:**

To assess the early real-world clinical outcomes regarding safety and efficacy after ranizurel administration.

**Methods:**

A retrospective, consecutive, interventional, uncontrolled, multi-centre study was conducted incorporating data from four centres in India. 22 eyes with variable indications were included and all patients were treated with at least one intravitreal injection of ranizurel 0.5 mg between January 2021 and April 2021. Each patient underwent best-corrected visual acuity (BCVA) measurement with a Snellen chart (converted to LogMAR for analysis), central subfield thickness (CST) analysis with spectral-domain optical coherence tomography (SD-OCT) and intraocular pressure (IOP) measurement along with complete ophthalmic examination at baseline and the last follow-up for evaluation of adverse events after ranizurel injection.

**Results:**

– None of the sites reported any signs of inflammation, vasculitis or any other ocular or systemic adverse effects in any of the cases. Mean BCVA at baseline was 0.48 ± 0.26 LogMAR (20/63) which improved significantly 0.26 ± 0.28 (20/40) at the last follow-up. (p = 0.001) Mean CST at baseline was 448.4 ± 122.9 μm which improved significantly to 328.3 ± 89.9 μm. (p = 0.001).

**Conclusion:**

– The early real-world data from this limited series indicates that ranizurel is a safe alternative biologic for patients who were treatment-naive and in those who had undergone prior treatment with other anti-VEGF agents.

Biologics targeting vascular endothelial growth factors (anti-VEGF) have revolutionized the treatment and outcomes of retinal vascular pathologies.^1^ However, the financial burden is found to be the major cause of patient non-compliance to monthly dosing regimens. Biosimilars were introduced to address this setback without any compromise on the safety or efficacy. Biosimilars in essence are similar to the innovator biologic or reference product with similar efficacy and safety profile and provide a cost-benefit of 20–30% compared to the reference molecules.^2^ The perception of biosimilars being generic drugs with poor safety and efficacy though has produced a nocebo effect. This has been scientifically disproved by the high safety profile of Razumab (Intas Pharmaceutical Ltd, Ahmedabad, India), the world's first biosimilar for ophthalmic use.^3^, ^4^, ^5^, ^6^, ^7^, ^8^, ^9^ Initial batches of razumab had shown immunogenicity which perpetuated the nocebo effect in the fraternity. However, the cause was identified and after the change in the manufacturing process, no further immunogenicity reactions are noted.

With this insight, we wanted to share our early experience with the initial batches of the drug about the safety of RanizuRel (Reliance Life Sciences, Mumbai, India) which is the second biosimilar of ranibizumab that has been approved by the drug controller general of India (DCGI) in the recent past.^10^ Recently the phase 3 trial data has been published.^11^ To the best of our knowledge, real-world clinical data of ranizurel use has not yet been reported. Here, we report the early clinical outcomes regarding safety and efficacy after ranizurel administration.

A retrospective, consecutive, interventional, uncontrolled, multi-centre study was conducted incorporating data from four centres in India. Institutional Review Board approval was obtained at each participating centre and the investigators adhered to the tenets of the Declaration of Helsinki. Consent was obtained from patients to use the data for research purpose. However, this report does not contain any personal identifying information. All patients were treated with at least one intravitreal injection of ranizurel 0.5 mg between January 2021 and April 2021. A minimum of 4-weeks of follow-up was required to be included in the study. The approved indications for the use of ranibizumab, the innovator molecule were considered for ranizurel administration which included retinal vein occlusions {(branch retinal vein occlusion (BRVO), non-ischemic central retinal vein occlusion (NICRVO) and hemi retinal vein occlusion (HRVO)}, diabetic macular edema (DME) and neovascular–age-related macular degeneration (n-AMD). Structural changes due to diseases other than the ones mentioned above and patients with vitreoretinal interface diseases were excluded. Each patient underwent best-corrected visual acuity (BCVA) measurement with a Snellen chart (converted to LogMAR for analysis), central subfield thickness (CST) with spectral-domain optical coherence tomography (SD-OCT) and intraocular pressure (IOP) measurement along with complete ophthalmic examination at the baseline and the last follow-up after ranizurel injection. Baseline and follow-up examinations included a thorough slit-lamp and dilated fundus evaluation for any adverse events including but not limited to inflammation, vasculitis, and haemorrhage. Descriptive statistics including mean, standard deviation (SD), median and range were calculated for continuous variables. Related Samples Wilcoxon Signed Rank Test was used to measure the differences between pre and post-injection values as the data distribution was non-parametric. Adverse events were reported in absolute numbers and frequencies.

22 eyes of 22 patients were included in this study which comprised of 11 DME, 5 BRVO, 1 NICRVO, 1 HRVO and 4 n-AMD eyes. The patients received a total of 34 injections. The mean age was 64.0 ± 8.5 (Range 45–74) years and 54.5% were males. The mean follow-up period was 8.3 ± 7.9 weeks after the first injection of ranizurel. Thirteen eyes received a single injection of ranizurel, 6 eyes (BRVO and n-AMD) received 2 injections and 3 eyes {DME^2^ and n-AMD^1^} had 3 injections. Eleven eyes were treatment naïve. All the other eight eyes were treated with other intravitreal anti-VEGFs (bevacizumab or ranibizumab or both). In addition to ranizurel, two eyes with DME were treated with focal laser, one eye with BRVO was treated with sectoral pan-retinal photocoagulation and one eye with DME with intravitreal dexamethasone implant (Allergan, Inc Irvine, Ca, USA). The median number of anti-VEGF injections in previously treated cases was 4 (Range 1–12). Four eyes had received ranibizumab, 2 eyes had received both ranibizumab and bevacizumab (Genentech, South San Francisco, CA, USA) and five eyes had received only bevacizumab. Immediate data before the first ranizurel injection was considered as the baseline, and the subsequent data after ranizurel injection were included in the analysis.

## 1 Key outcomes

### 1.1 Safety

The occurrence of ocular or systemic adverse events was noted at each follow-up visit after ranizurel treatment. In particular, the signs of anterior and posterior segment inflammation were evaluated. In this study, none of the sites reported any signs of inflammation, vasculitis, or any other ocular or systemic adverse effects in any of their cases.

### 1.2 Visual acuity

Overall mean BCVA at baseline was 0.48 ± 0.26 LogMAR (20/63) and was 0.26 ±0 .28 (20/40) at the last follow-up (p = 0.001). The changes in BCVA in various subgroups have been depicted in Table 1.

**Table 1:** Changes in BCVA and CST from baseline to the last followup.

Parameters	Indication	Baseline	Last-Follow-up	p-value
BCVA	Cumulative (n = 19)	0.48 ± 0.26 logMAR	0.26 ± 0.28 logMAR	0.001^a^
	DME (n = 9)	0.50 ± 0.27 logMAR	0.30 ± 0.29 logMAR	0.004^a^
	RVO (n = 6)	0.41 ± 0.18 logMAR	0.14 ± 0.22 logMAR	0.027^a^
	nAMD (n = 4)	0.55 ± 0.41 logMAR	0.38 ± 0.35 logMAR	0.102
CST	Cumulative (n = 19)	448.4 ± 122.9 μ	328.3 ± 89.9 μ	0.001^a^
	DME (n = 9)	473.4 ± 162.3 μ	339.5 ± 96.9 μ	0.003^a^
	RVO (n = 6)	413.1 ± 74.4 μ	299.4 ± 83.7 μ	0.028^a^
	nAMD (n = 4)	441.25 ± 41.6 μ	348.0 ± 91.3 μ	0.109

BCVA: Best Corrected Visual Acuity, CST: Central Subfield Thickness, DME: Diabetic Macular Edema, RVO: Retinal venous occlusion, nAMD: Neovascular Age-related Macular Degeneration.

a p-value <0.05 is clinically significant.

### 1.3 Disease **activity** (Fig. 1)

Mean CST at baseline was 448.4 ± 122.9 μm which improved significantly to 328.31.7 ± 89.9 μm. (p = 0.001). The variation in CST in various indications is tabulated in Table 1.

**Fig. 1:**
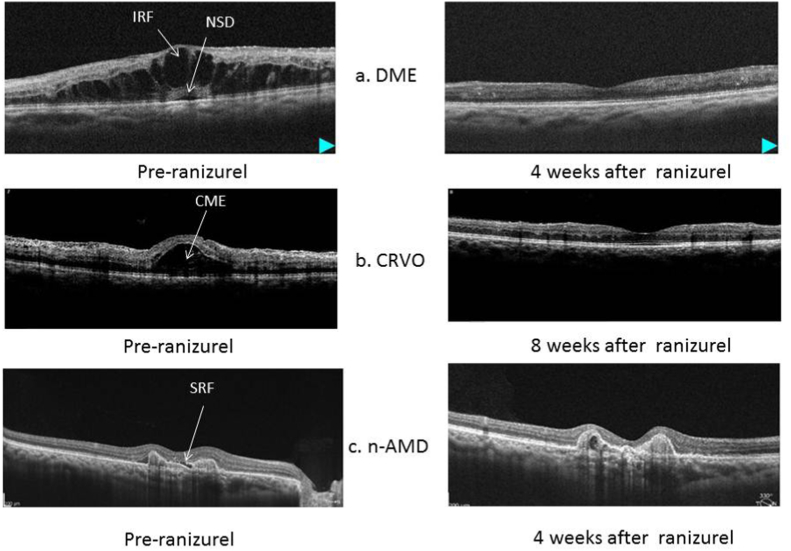
(a–c). a- Representative case of DME with IRF and NSD at baseline and complete resolution after 4 weeks of ranizurel injectionb - Representative case of CRVO with CME at baseline and complete resolution of CME after 8 weeks of ranizurel injectionc - Representative case of n -AMD with SRF at baseline and complete resolution of SRF after 4 weeks of ranizurel injection.

To summarize, early real-world data in this limited series demonstrated that ranizurel was safe in patients who were treatment-naive and in those who undergone prior treatment with other anti-VEGF agents. Limited data also showed improved BCVA and CST in RVO and DME cases and stable BCVA and CMT in nAMD cases, but further studies are required to comment robustly on its efficacy.

Immunogenicity has been a challenging area concerning biologics and biosimilars lately.^12^ Brolucizumab demonstrated immunogenicity in the real world and abicipar could not receive US-FDA approval predominantly due to safety concerns caused by immunogenicity.^13^^,^^14^ The first biosimilar of ranibizumab had also shown immunogenicity in the initial few batches.^12^ However, the company had taken corrective steps by changing the standard references for endotoxin levels, and now the drug is being widely used in India and many published studies have proven its safety.^4^, ^5^, ^6^, ^7^, ^8^, ^9^ Switching to a biosimilar from the reference biologic has also been discussed by the regulatory authorities.^15^ We have found no signs of immunogenicity in response to switching from the reference ranibizumab to the first biosimilar of ranibizumab (Razumab) approved in India.^16^

In this study, we did not find any case of anterior or posterior segment inflammation with the ranizurel. No systemic adverse events also were not noted. In the early real-world experience, the molecule appears to be safe, but a long term follow-up with a larger sample size will be required to effectively identify more rare adverse events. Hence, these results are just indicative. Studies with larger sample size and longer duration of follow-up with a comparison arm with the reference biologic or another biosimilar will be important to better understand the anatomic efficacy and durability benefits of ranizurel.

## Acknowledgement and disclosures

No funding or grant support for this manuscript.

Dr Kuppermann acknowledges an unrestricted grant from 10.13039/100001818Research to Prevent Blindness to the Gavin Herbert Eye Institute at the 10.13039/100008476University of California, Irvine.

## Authorship

All authors attest that they meet the current ICMJE criteria for Authorship.

## Declaration of competing interest

Dr Ashish: CONSULTANT: for Novartis, Allergan, Bayer and Intas.

Dr Nilesh Kumar: Investigator: Novartis.

Dr Nikulaa P: Investigator: Novartis.

Baruch D Kuppermann: CLINICAL RESEARCH: Alcon, Alimera, Allegro, Allergan, Apellis, Clearside, Genentech, GSK, Ionis, jCyte, Novartis, Regeneron, ThromboGenics; CONSULTANT: Alimera, Allegro, Allergan, Cell Care, Dose, Eyedaptic, Galimedix, Genentech, Glaukos, Interface Biologics, jCyte, Novartis, Ophthotech, Regeneron, Revana, Theravance Biopharma.

The following authors have no financial disclosures.

Dr Jayshree Arunaprakash: None.

Dr Ashraya Nayaka: None.

Dr Atheeshwar Das: None.
